# Experimental data on morphological characterization of chars from coal and bagasse blends

**DOI:** 10.1016/j.dib.2020.105358

**Published:** 2020-03-02

**Authors:** Edward Garcia, Juan Barraza-Burgos, Juan Guerrero, María Trujillo, Edward Lester, Orla Williams

**Affiliations:** a Facultad de Ingeniería, Universidad Del Valle, Ciudad Universitaria Meléndez, Calle 13 # 100-00, A. A. 25360, Cali, Colombia; b Faculty of Engineering, University of Nottingham, University Park, NG7 2RD Nottingham, United Kingdom

**Keywords:** Char, Morphology, Coal, Bagasse, Blends, Reactivity

## Abstract

Morphological characterization of chars from coal and bagasse plays an important role in both the burning efficiency and intrinsic reactivity of chars, during a combustion process [1], [2]. In this work, abundant data on the morphology of chars produced from coal and bagasse blends are presented. Char synthesis was performed varying the temperature (900, 1000 and 1100 °C) and bagasse proportion feeding (0, 25, 50, 75 and 100% w/w) in the pyrolysis reaction. Proximate, ultimate, petrographic and vitrinite reflectance of raw coal and bagasse are presented. Char morphology is classified into three groups -- thin walls, thick walls, and solid particles--, and results are exhibited. The data set is a comprehensive source for advancing in a further understanding of char's morphology from coal-bagasse blends.

**Table:** Specifications Table

Subject	Chemical Engineering::Chemical Engineering (General)
Specific subject area	Energy and material science
Type of data	Figures and tables
How data were acquired	Data were obtained by proximate analysis (LECO TGA-601), ultimate analysis (LECO TruSpec CHN), petrography analysis (Olympus BX-51), morphological microscopy (Nikon LV-100), SEM microscopy (Jeol JSM6490LV). Chars from coal and bagasse blends were obtained in an entrainment reactor using nitrogen at three pyrolysis temperatures (900, 1000 and 1100 °C) and three blend proportions (0, 25, 50, 75 and 100% w/w).
Data format	Raw and analyzed data
Parameters for data collection	Original coals and bagasse samples for char's synthesis were characterized by proximate, Heat High Value (HHV), ultimate, sulphur, petrography and vitrinite reflectance analysis. Char´s morphology of coals, bagasse and blends were characterized according to the thick and thin network morphologies, thick and thin spheres, pore mix, dense mixture, inert, solids and filaments.
Description of data collection	Raw data of proximate, HHV, ultimate and sulphur analysis (Table 1) were made according to ASTM D 7582-10, ASTM D5865-11^a^, ASTM D5378-08, ASTM D4239 – 12 methods respectively. Raw data of petrography and vitrinite reflectance (Table 2) according to ASTM D2799–1 and ASTM D2798-11a. Raw data of char`s morphological classification (Table 3), Char`s morphology according to each morphology type (Table 4). Analyzed data of char´s morphology frequency from original coal and bagasse as a function of the temperature (Fig. 1) and analyzed data of char´s morphology frequency from coal-bagasse blend as a function of the temperature (Fig. 2, Fig. 3, Fig. 4).
Data source location	Char´s production and its morphological characterization were done in the Chemical Engineering School of the Universidad del Valle, (Cali, Colombia).
Data accessibility	Data are provided in this article.

**Table:** 

**Value of the Data** • The char´s morphology raw data is useful to estimate coal-bagasse blends reactivity at larger scales processes, such as combustion and gasification. It is also a novelty data concerning the morphological characterization of chars from coal-bagasse blends. The raw data of proximate, ultimate, petrographic and SEM analyses can be used to evaluate the quality of coal-bagasse blends. • The data are relevant for researchers seeking for a classification system to characterize chars from coal-bagasse blends.| • The data can be used for further experiments such as thermogravimetric analysis (TGA), which can correlate char´s morphology vs. characteristic reactivity temperatures. • The data of changes in char´s morphology from coal-bagasse blends impact thermochemical conversion.

## 1 Data description

Data presented in this work describes the production and characterization of chars derived from coal of three Colombian regions (Cundinamarca, Antioquia and Valle States) and bagasse obtained from a sugar refinery mill located at the South West Colombian region. The data corresponds to the synthesis of char at different values of temperature (900, 1000 and 1100 °C) and three blend proportions (0, 25, 50, 75 and 100% w/w) in the pyrolysis reaction.

Table 1 presents the proximate and ultimate analysis of coals and bagasse. Table 2 shows the petrography and vitrinite reflectance (% v/v mineral matter free, mmf). Table 3 displays the char morphological classification of coals and bagasse [4]. Table 4 shows the char`s morphology according to each type of char, where the used nomenclature is the following: the first letter C: Char; followed by the letter A, C, V or B indicating the origin of the material, Antioquia, Valle, Cundinamarca or Bagasse. The following character 9, 10, 11 stands for the pyrolysis temperature of 900, 1000 and 1100 °C. The numbers 00, 25, 50, 75, 100, indicates the percentage of bagasse in the blend. For example, C-V-9-25 represents a char from Valle coal, obtained at 900 °C with 25% w/w of bagasse.

**Table 1:** Proximate and ultimate analysis of original coals and bagasse.

	Antioquia	Cundinamarca	Valle	Bagasse
*Proximate, % w/w, dry basis, db*				
Ash	9.56	11.68	31.62	31.02
Volatile matter	44.03	37.73	29.62	60.37
Fixed carbon (calculated)	46.42	50.60	38.76	8.62
*Ultimate, % w/w, dry basis mineral matter free (dbmmf)*				
C	77.67	84.36	76.94	56.24
H	4.22	5.60	5.79	6.32
N	1.60	1.99	1.41	0.50
S	0.99	1.71	5.12	0.09
O (by difference)	15.51	6.35	10.74	36.86
Heat value, Btu/lb	11406	13003	9450	6091

Source: Authors – Raw data.

**Table 2:** Petrography and vitrinite reflectance of coals (% v/v mineral matter free, mmf).

Maceral	Antioquia	Cundinamarca	Valle
Vitrinite	81.6	65.6	86.5
Liptinite	16.1	9.7	9.7
Inertinite	2.3	24.8	3.8
Vitrinite reflectance (Ro, %)	0.45	0.84	0.84

Source: Authors– Raw data.

**Table 3:**
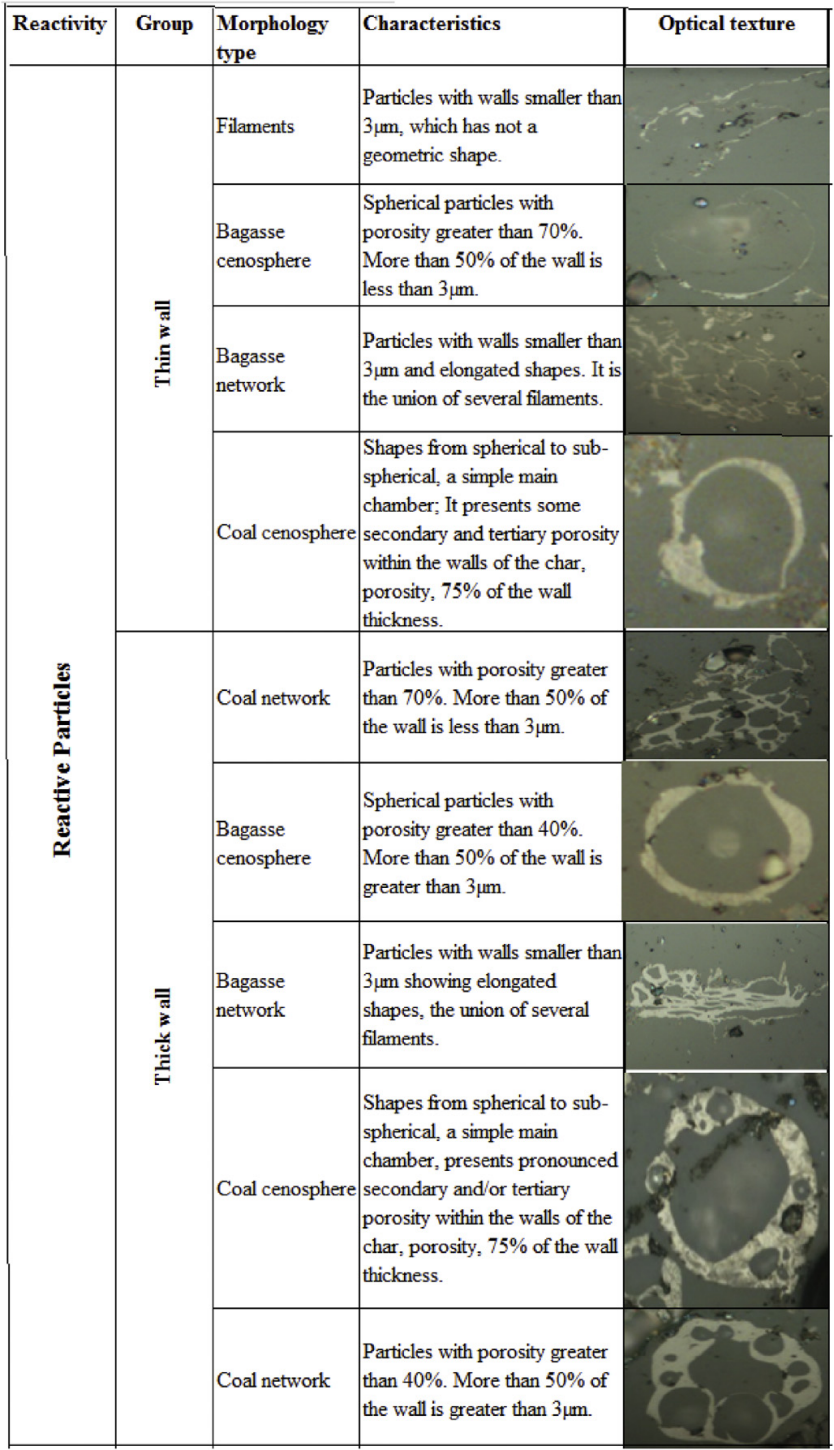

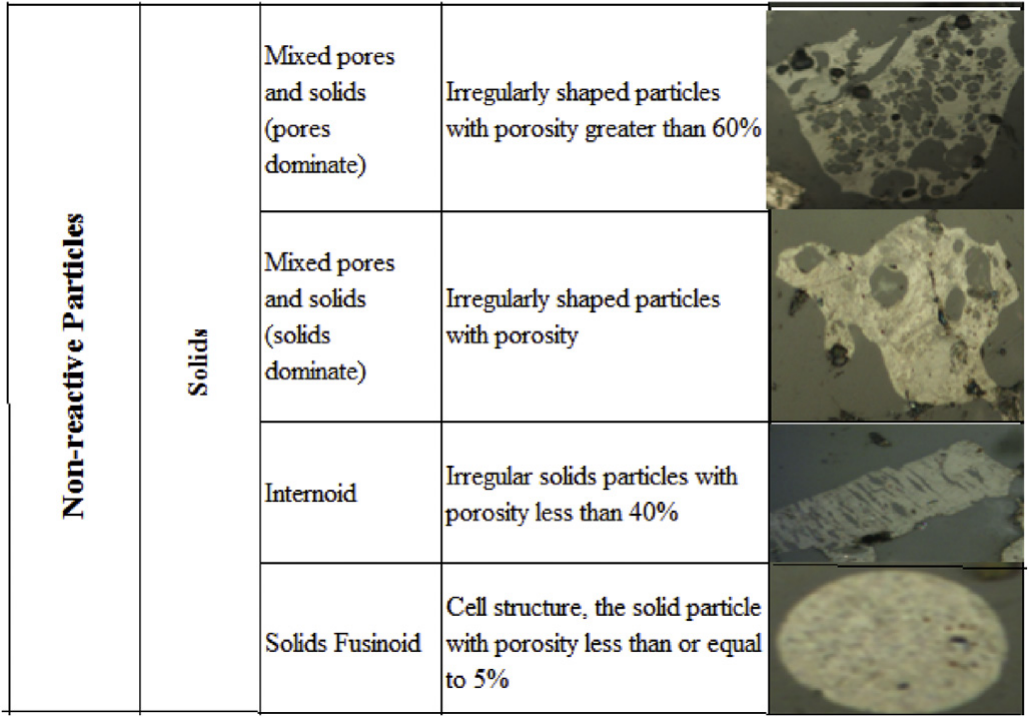
Char`s morphological classification of coals and bagasse.

Source: Authors– Raw data.

**Table 4:** Char`s morphology according to each morphology type.

Sample	Ítem	Bagasse					Coal							
		Filaments	Thin spheres	Thick spheres	Thin network	Thick network	Thin spheres	Thick spheres	Thin network	Thick network	Mix porous	Mix dense	Inertoid	Solids
CA900	Total	0	0	0	0	0	3	23.5	8.5	106.5	101	79.5	53	29
	%	0.00	0.00	0.00	0.00	0.00	0.74	5.82	2.10	26.36	25.00	19.68	13.12	7.18
CA925	Total	2.5	3	0	6.5	3	4.5	13.5	3.5	90.5	70.5	101	99.5	40.5
	%	0.57	0.68	0.00	1.48	0.68	1.03	3.08	0.80	20.64	16.08	23.03	22.69	9.24
CA950	Total	2.5	0.5	2	11	16	2	16.5	0.5	64.5	63	83	82.5	75.5
	%	0.60	0.12	0.48	2.62	3.81	0.48	3.93	0.12	15.38	15.02	19.79	19.67	18.00
CA975	Total	28	6	8.5	41.5	73	1	26	3	79	75	25	14.5	25.5
	%	6.90	1.48	2.09	10.22	17.98	0.25	6.40	0.74	19.46	18.47	6.16	3.57	6.28
CC900	Total	0	0	0	0	0	53	61	68	134	26	19	32	7
	%	0.00	0.00	0.00	0.00	0.00	13.25	15.25	17.00	33.50	6.50	4.75	8.00	1.75
CC925	Total	2	5	0	46	21	30	68	52	86	25	15	37	16
	%	0.50	1.24	0.00	11.41	5.21	7.44	16.87	12.90	21.34	6.20	3.72	9.18	3.97
CC950	Total	3	6	2	60	36	23	45	20	76	20	15	61	39
	%	0.74	1.48	0.49	14.78	8.87	5.67	11.08	4.93	18.72	4.93	3.69	15.02	9.61
CC975	Total	0	12	14	163	62	2	37	2	33	20	6	23	29
	%	0.00	2.98	3.47	40.45	15.38	0.50	9.18	0.50	8.19	4.96	1.49	5.71	7.20
CV950	Total	50.5	19.5	3.5	66.5	26.5	22	36	31.5	58.5	31.5	37	20	16.5
	%	12	5	1	16	6	5	9	8	14	8	9	5	4
CV975	Total	58	53	9.5	199	17.5	4.5	3	4	7	7.5	18	11.5	7
	%	15	13	2	50	4	1	1	1	2	2	5	3	2
CB9100	Total	42	15	7	275	54	0	0	0	0	0	0	0	0
	%	10.69	3.82	1.78	69.97	13.74	0.00	0.00	0.00	0.00	0.00	0.00	0.00	0.00
CA1000	Total	0	0	0	0	0	19	12	105	60	48	82	57	17
	%	0.00	0.00	0.00	0.00	0.00	4.75	3.00	26.25	15.00	12.00	20.50	14.25	4.25
CA1025	Total	3	2	0	50	6	12	15	44	58	58	69	68	15
	%	0.75	0.50	0.00	12.50	1.50	3.00	3.75	11.00	14.50	14.50	17.25	17.00	3.75
CA1050	Total	3	3	0	79	7	15	12	52	60	67	52	39	12
	%	0.75	0.75	0.00	19.75	1.75	3.75	3.00	13.00	15.00	16.75	13.00	9.75	3.00
CA1075	Total	21	5	0	211	15	6	7	38	29	23	29	27	4
	%	5.25	1.25	0.00	52.75	3.75	1.50	1.75	9.50	7.25	5.75	7.25	6.75	1.00
CC1000	Total	0	0	0	0	0	29	74	98	110	24	24	21	21
	%	0.00	0.00	0.00	0.00	0.00	7.23	18.45	24.44	27.43	5.99	5.99	5.24	5.24
CC1025	Total	0	0	0	29	16	35	81	37	104	40	25	23	10
	%	0.00	0.00	0.00	7.25	4.00	8.75	20.25	9.25	26.00	10.00	6.25	5.75	2.50
CC1050	Total	2	5	3	149	15	18	76	30	43	21	19	9	10
	%	0.50	1.25	0.75	37.25	3.75	4.50	19.00	7.50	10.75	5.25	4.75	2.25	2.50
CC1075	Total	8	3	1	206	15	14	38	31	42	16	14	9	3
	%	2.00	0.75	0.25	51.50	3.75	3.50	9.50	7.75	10.50	4.00	3.50	2.25	0.75
CV1000	Total	0	0	0	0	0	18	67	148	127	20	6	6	8
	%	0.00	0.00	0.00	0.00	0.00	4.50	16.75	37.00	31.75	5.00	1.50	1.50	2.00
CV1025	Total	3	0	0	26	10	12	66	115	105	30	11	6	18
	%	0.75	0.00	0.00	6.47	2.49	2.99	16.42	28.61	26.12	7.46	2.74	1.49	4.48
CV1050	Total	5	3	0	60	14	22	62	71	102	20	14	8	19
	%	1.25	0.75	0.00	15.00	3.50	5.50	15.50	17.75	25.50	5.00	3.50	2.00	4.75
CV1075	Total	9	11	5	169	19	27	48	38	40	26	4	8	4
	%	2.21	2.70	1.23	41.42	4.66	6.62	11.76	9.31	9.80	6.37	0.98	1.96	0.98
CB10100	Total	42	15	7	275	54	0	0	0	0	0	0	0	0
	%	10.50	3.75	1.75	68.75	13.50	0.00	0.00	0.00	0.00	0.00	0.00	0.00	0.00
CA1100	Total	0	0	0	0	0	11	19	97	73	83	58	40	19
	%	0.00	0.00	0.00	0.00	0.00	2.75	4.75	24.25	18.25	20.75	14.50	10.00	4.75
CA1125	Total	2	3	0	36	10	10	10	69	63	69	77	51	3
	%	0.50	0.74	0.00	8.93	2.48	2.48	2.48	17.12	15.63	17.12	19.11	12.66	0.74
CA1150	Total	3	11	4	110	7	10	17	30	52	61	47	48	8
	%	0.74	2.73	0.99	27.30	1.74	2.48	4.22	7.44	12.90	15.14	11.66	11.91	1.99
CA1175	Total	15	13	8	149	28	9	7	19	36	42	46	24	4
	%	3.72	3.23	1.99	36.97	6.95	2.23	1.74	4.71	8.93	10.42	11.41	5.96	0.99
CC1100	Total	0	0	0	0	0	37	69	61	161	23	20	27	2
	%	0.00	0.00	0.00	0.00	0.00	9.25	17.25	15.25	40.25	5.75	5.00	6.75	0.50
CC1125	Total	11	1	0	41	32	25	50	61	81	24	16	19	26
	%	2.84	0.26	0.00	10.59	8.27	6.46	12.92	15.76	20.93	6.20	4.13	4.91	6.72
CC1150	Total	11	6	3	80	27	23	34	58	78	23	15	34	29
	%	2.61	1.43	0.71	19.00	6.41	5.46	8.08	13.78	18.53	5.46	3.56	8.08	6.89
CC1175	Total	15	3	2	88	33	1	11	27	58	24	11	15	12
	%	5.00	1.00	0.67	29.33	11.00	0.33	3.67	9.00	19.33	8.00	3.67	5.00	4.00
CV1100	Total	0	0	0	0	0	20	116	62	138	28	18	10	12
	%	0.00	0.00	0.00	0.00	0.00	4.95	28.71	15.35	34.16	6.93	4.46	2.48	2.97
CV1125	Total	7	4	4	38	22	11	80	55	92	30	32	13	17
	%	1.73	0.99	0.99	9.38	5.43	2.72	19.75	13.58	22.72	7.41	7.90	3.21	4.20
CV1150	Total	20	10	1	92	16	3	10	20	69	34	37	78	14
	%	4.95	2.48	0.25	22.77	3.96	0.74	2.48	4.95	17.08	8.42	9.16	19.31	3.47
CV1175	Total	21	13	1	144	44	4	11	28	30	27	43	32	3
	%	5.24	3.24	0.25	35.91	10.97	1.00	2.74	6.98	7.48	6.73	10.72	7.98	0.75
CB11100	Total	34	24	7	279	54	0	0	0	0	0	0	0	0
	%	8.44	5.96	1.74	69.23	13.40	0.00	0.00	0.00	0.00	0.00	0.00	0.00	0.00

Source: Authors– Raw data.

**Fig. 1:**
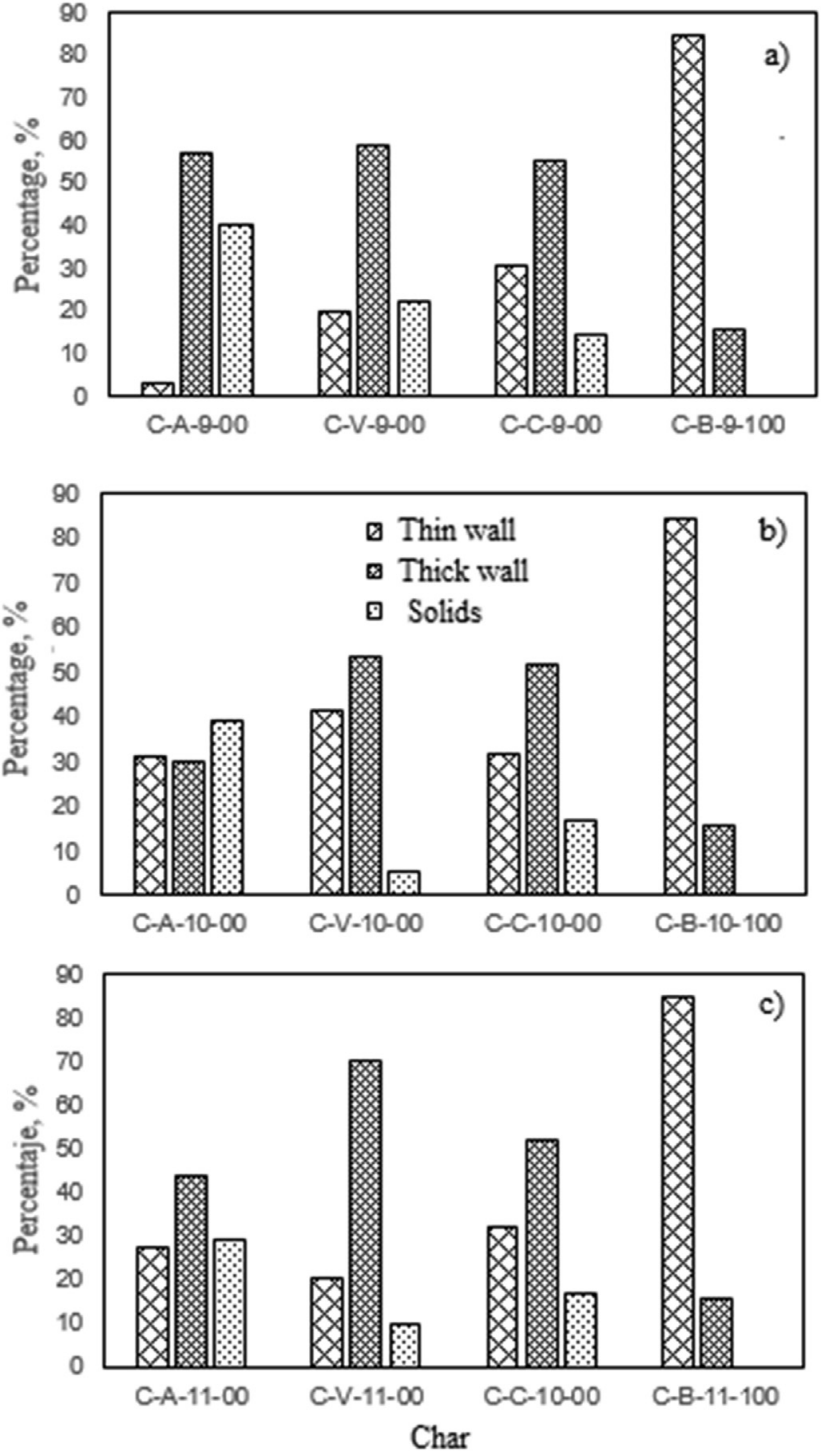
Char´s morphology frequency from original coal and bagasse as a function of the temperature, a) 900 °C, b) 1000 °C, c) 1100 °C. Source: Authors– analyzed data.

**Fig. 2:**
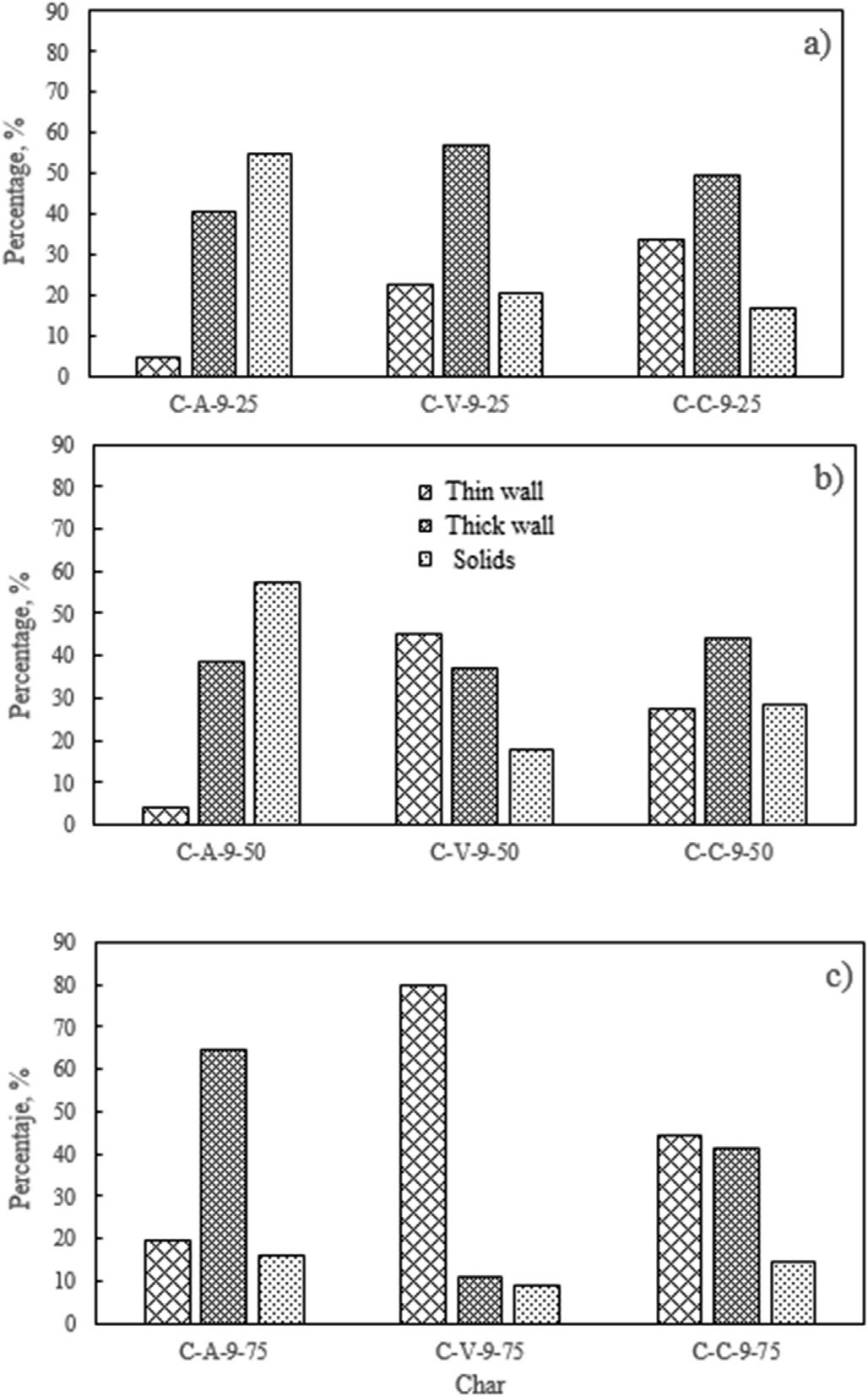
Char´s morphology frequency from coal-bagasse blend at 900 °C. a) 25%, b) 50%, c) 75%. Source: Authors– analyzed data.

**Fig. 3:**
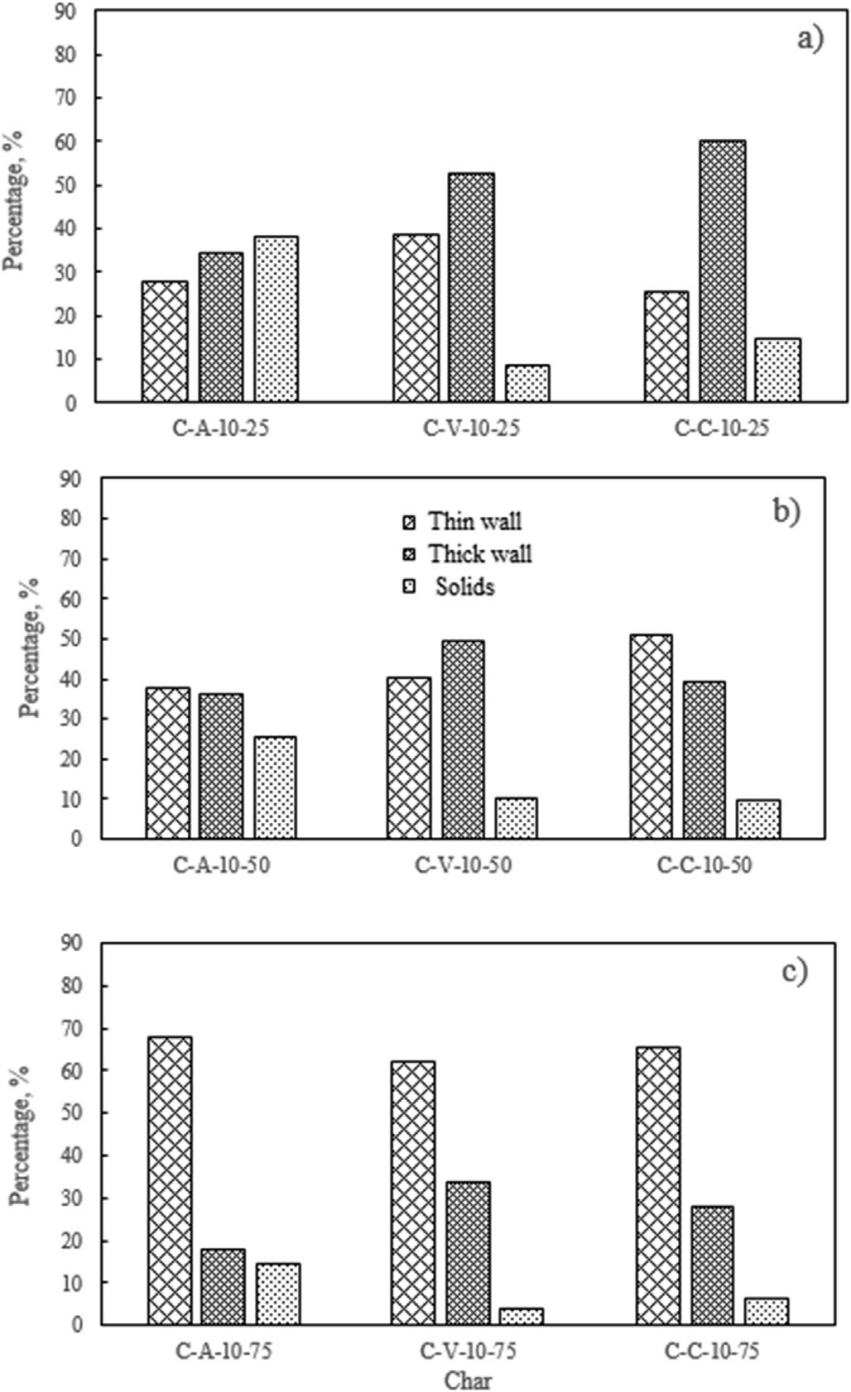
Char´s morphology frequency from coal-bagasse blend at 1000 °C. a) 25%, b) 50%, c) 75%. Source: Authors– analyzed data.

**Fig. 4:**
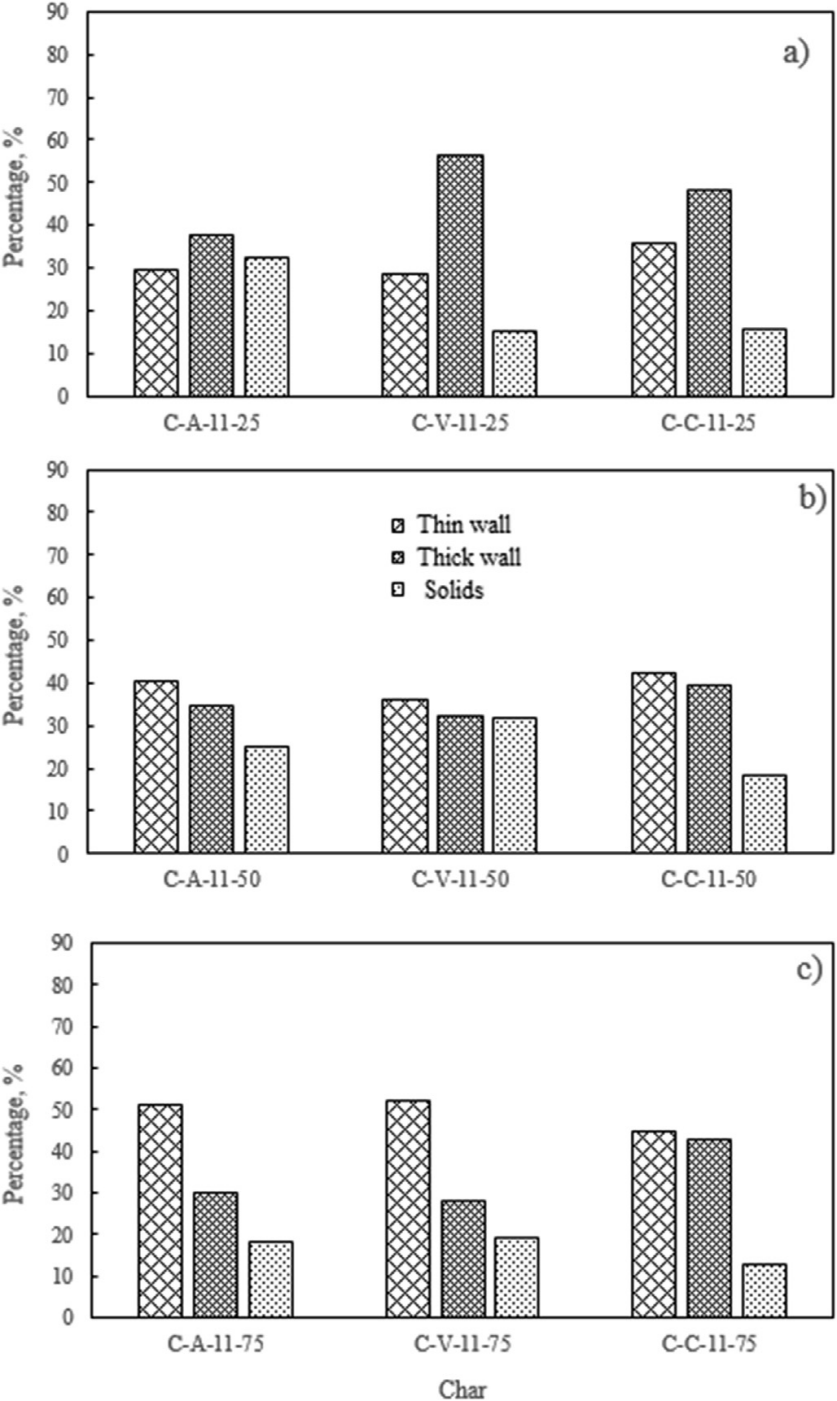
Char´s morphology frequency from coal-bagasse blend at 1100 °C. a) 25%, b) 50%, c) 75%. Source: Authors– analyzed data.

**Fig. 5:**
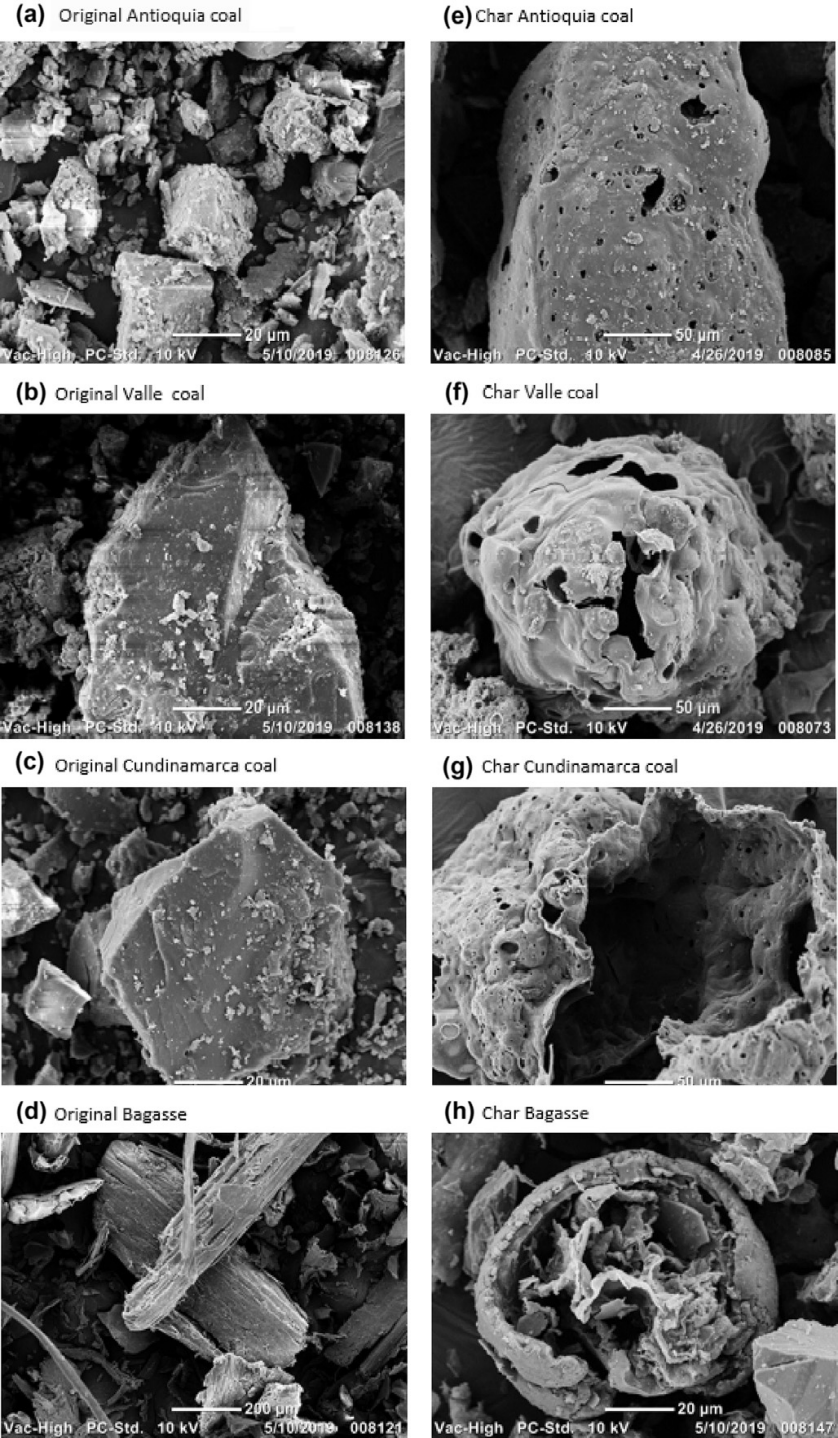
SEM morphology of original and char samples produced at 900 °C. Source: Authors– Raw data.

Regarding the included figures, Fig. 1 shows the morphology frequency of chars from the original coals and bagasse as a function of temperatures 900, 1000 and 1100 °C. Fig. 2, Fig. 3, Fig. 4 show the morphology frequency of chars from coals and bagasse blends (25, 50 and 75% w/w) at temperatures 900, 1000 and 1100 °C. Finally, Fig. 5 portrays the morphology of the original and char samples of the three coals and bagasse through the SEM technique.

## 2 Experimental design, materials, and methods

### 2.1 Synthesis of Chars

**Fig. 6:**
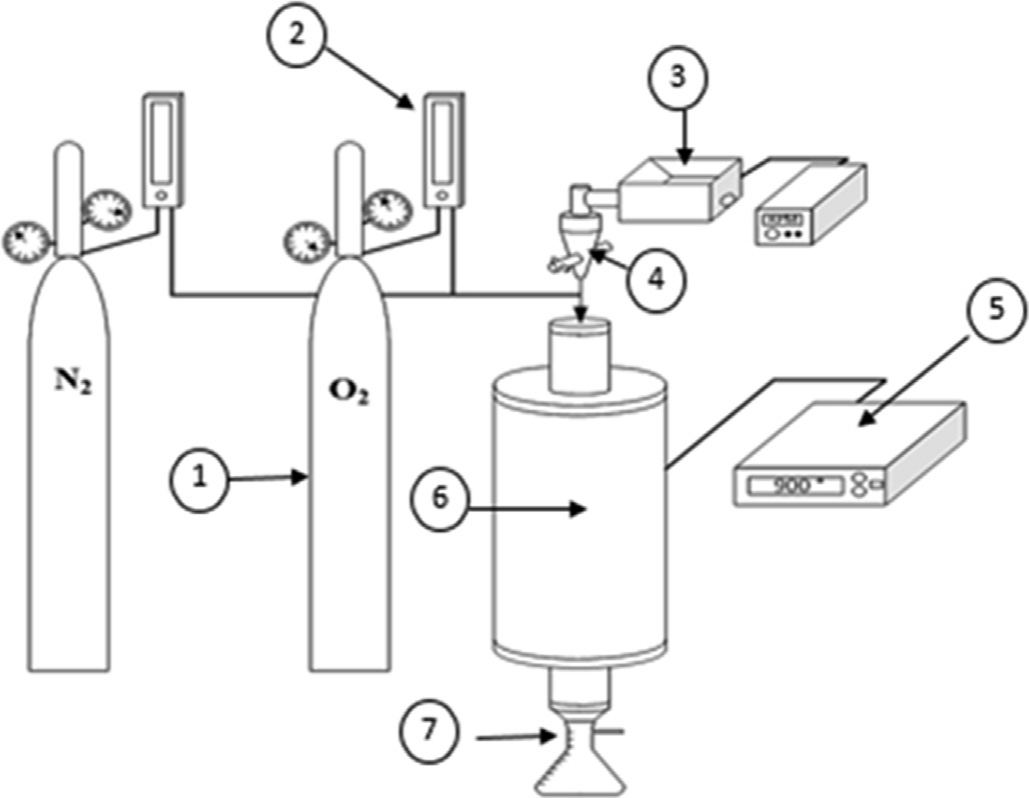
Process diagram to obtain chars from coal and bagasse blends. Source: Authors.

Fig. 6 shows a process diagram for obtaining chars. This system consists of a vertical tubular entrainment reactor [6] with an isothermal zone of 180 mm. The quartz tube has a length of 900 mm and an external diameter of 60 mm, with a wall thickness of 1 mm. The reactor is heated with a Carbolite STF 18 tubular oven. The reactor uses an inert atmosphere controlled with nitrogen and oxygen flows, with a total flow of 88 ml/s containing 5% v/v oxygen, to avoid the tars condensation. The system for obtaining chars is composed of a temperature controller [5], a feed system for displacement screw-type samples [[3], [4]], a char and gas separator (7), and a feed system for N_2_ and O_2_ gases [1] with their respective flow meters [2].

### 2.2 Experimental design

A randomized complete experimental block design was used for obtaining char samples, taking into account three factors: coal origin, the percentage of bagasse in the coal-bagasse blends and pyrolysis temperatures. Coal origin: in this investigation, three carbons from different regions of Colombia (Cundinamarca, Antioquia and Valle States) were analyzed since the morphology of chars is related to the range and composition of the original sample. The percentage of bagasse: blends of coal-bagasse were made in proportions of 0, 25, 50, 75 and 100% w/w of bagasse. Pyrolysis temperatures: three temperatures were used, 900, 1000 and 1100 °C. The particle size of 250 μm was used for all samples. The samples were fed employing a feeding system consisting of a hopper equipped with an endless screw driven by an electric motor with speed variation. In total 50 g were pyrolyzed per run over a period of 2 hours. The combinations of the different levels of the three factors and the replicas gave a total of 90 pyrolysis experiments, which were carried out randomly. The obtained chars were stored hermetically for later analysis.

### 2.3 Characterization of coals, bagasse, and chars

#### 2.3.1 Proximate analysis

The contents of volatile matter, ash, fixed carbon, and moisture were determined by recording the weight loss when the temperature increases under a controlled atmosphere, using a LECO TGA-601 thermogravimetric balance. All analyses were performed by duplicate with an error rate of less than 1%, following the methodology proposed by ASTM D 7582-10. The calorific value was obtained by burning a gram of sample in a LECO AC600 calorimeter following the procedure of ASTM D5865-11^a^.

#### 2.3.2 Ultimate analysis

For the determination of Carbon (C), Hydrogen (H) and Nitrogen (N), combustion at 950 ° C was carried out in an oxygen atmosphere of 1 g of sample. The oxidation reactions of these elements produce CO_2_, H_2_O and NOx gases. The last gas was reduced to elementary N, detected by thermal conductivity, while C and H were analyzed by infrared absorption. This procedure was performed in a TruSpec CHN LECO analyzer, using ASTM D5378-08. To determine the total sulphur content, a LECO S-144 Sulphur analyzer was used, following ASTM D4239-12. A sample of 1 g was introduced into a tubular furnace that was subsequently heated to 1350 ° C under an atmosphere oxidizing, producing the SO_2_ gas, which was measured in an infrared absorption detector. The oxygen was calculated by difference.

#### 2.3.3 Petrography analysis

For the determination of the coal maceral composition, specimens were made by embedding a sample of approximately 0.4 g in epoxy resin powder, mixed with Buehler brand liquid hardener. The sample was hardened, by a curing process for 24 hours. The specimens were roughened with 400, 600 and 1200 grains of sands and then polished with metallographic cloths, using alumina suspensions of 1.0, 0.3 and 0.05 μm at 200 rpm in a Struers Labopol-5 brand polishing machine until the specimens with their surface were obtained without any scratches. The prepared samples were analyzed in a Nikon LV100 metallographic microscope using a 50X air objective to determine the maceral composition by ASTM D2799-1. The 3 most important microscopic components of coal were identified as inertinite, liptinite, and vitrinite; and from this analysis, the maceral volume percentage was obtained.

#### 2.2.4 Vitrinite reflectance analysis

The vitrinite reflectance was performed on an Olympus BX-51 microscope coupled to a J&M MSP 200 spectrophotometer, using reflected light, polarized monochromatic, with a 50X oil objective. Initially, the system was calibrated using 5 standard patterns of known reflectance. The average reflectance was determined following the procedure of the ASTM D2798-11a***.***

#### 2.2.5 Morphology analysis

For this purpose, specimens were made by mixing 0.2 g of carbonized with LECO brand epoxy resin in a one-inch diameter mould. The mixture was cured for 24 hours, then extracted from the mould and subjected to a sanding and polishing process in the Struers Labopol-5 brand polishing machine, at 200 rpm, using 400, 600 and 1200 grain of sands; and for polishing, alumina suspensions of 1.0, 0.3, 0.05 μm. The polishing times exerted varied at each stage depending on the proportion of bagasse in the sample. This is because biomass is a soft material and easy to erode, and therefore imperfections occur on its surface. The sanded and polished specimens were analyzed with a Nikon LV-100 metallographic microscope using reflected white light, with a 50X air objective. In this analysis, at least 400 particles were identified for each test. Each carbonized particle was classified according to Avila et al. [3], Lester et al. [4], and Sanabria and Castro [5]. Morphologies were identified according to their origin, coal or bagasse, with thick and thin network morphologies, thick and thin spheres, pore mix, dense mixture, inert, solids and filaments that are exclusive structures of bagasse particles. To evaluate the reactivity, morphologies were gathered into three groups [6]), in thin walls, thick walls and solid particles, where the first 2 are porous mixtures, and the last consists of mixtures dense, solid and inert.

#### 2.2.6 SEM analysis

A scanning electron microscope (Jeol JSM6490LV) was used, with a probe for chemical microanalysis (INCAPenta FETx3), with sample coating systems (gold and carbon) and with heating and cooling plates.
